# Validation of the standardization framework SSTR-RADS 1.0 for neuroendocrine tumors using the novel SSTR‑targeting peptide [^18^F]SiTATE

**DOI:** 10.1007/s00330-024-10788-3

**Published:** 2024-05-20

**Authors:** R. Ebner, A. Lohse, M. P. Fabritius, J. Rübenthaler, C. Wängler, B. Wängler, R. Schirrmacher, F. Völter, H. P. Schmid, L. M. Unterrainer, O. Öcal, A. Hinterberger, C. Spitzweg, C. J. Auernhammer, T. Geyer, J. Ricke, P. Bartenstein, A. Holzgreve, F. Grawe

**Affiliations:** 1 Department of Radiology, LMU University Hospital, LMU Munich, Munich, Germany; 2 Interdisciplinary Center of Neuroendocrine Tumors of the GastroEnteroPancreatic System (GEPNET-KUM, ENETS certified Center of Excellence), LMU University Hospital, LMU Munich, Munich, Germany; 3 Biomedical Chemistry, Clinic of Radiology and Nuclear Medicine, Medical Faculty Mannheim of Heidelberg University, Mannheim, Germany; 4 Molecular Imaging and Radiochemistry, Clinic of Radiology and Nuclear Medicine, Medical Faculty Mannheim of Heidelberg University, Mannheim, Germany; 5 Department of Oncology, Division of Oncological Imaging, University of Alberta, Edmonton, Canada; 6 Department of Nuclear Medicine, LMU University Hospital, LMU Munich, Munich, Germany; 7 DKFZ Hector Cancer Institute at the University Medical Center Mannheim, Heidelberg, Germany; 8 Department of Medicine IV, LMU University Hospital, LMU Munich, Munich, Germany; 9 Department of Clinical Radiology and Nuclear Medicine, University Medical Center Mannheim, Medical Faculty Mannheim, Heidelberg University, Mannheim, Germany

**Keywords:** Neuroendocrine tumors, Somatostatin, Molecular imaging, Positron emission tomography-computed tomography

## Abstract

**Objectives:**

Somatostatin receptor positron emission tomography/computed tomography (SSTR-PET/CT) using [^68^Ga]-labeled tracers is a widely used imaging modality for neuroendocrine tumors (NET). Recently, [^18^F]SiTATE, a SiFAlin tagged [Tyr3]-octreotate (TATE) PET tracer, has shown great potential due to favorable clinical characteristics. We aimed to evaluate the reproducibility of Somatostatin Receptor-Reporting and Data System 1.0 (SSTR-RADS 1.0) for structured interpretation and treatment planning of NET using [^18^F]SiTATE.

**Methods:**

Four readers assessed [^18^F]SiTATE-PET/CT of 95 patients according to the SSTR-RADS 1.0 criteria at two different time points. Each reader evaluated up to five target lesions per scan. The overall scan score and the decision on peptide receptor radionuclide therapy (PRRT) were considered. Inter- and intra-reader agreement was determined using the intraclass correlation coefficient (ICC).

**Results:**

The ICC analysis on the inter-reader agreement using SSTR-RADS 1.0 for identical target lesions (ICC ≥ 85%), overall scan score (ICC ≥ 90%), and the decision to recommend PRRT (ICC ≥ 85%) showed excellent agreement. However, significant differences were observed in recommending PRRT among experienced readers (ER) (*p* = 0.020) and inexperienced readers (IR) (*p* = 0.004). Compartment-based analysis demonstrated good to excellent inter-reader agreement for most organs (ICC ≥ 74%), except for lymph nodes (ICC ≥ 53%).

**Conclusion:**

SSTR-RADS 1.0 represents a highly reproducible and consistent framework system for stratifying SSTR-targeted PET/CT scans, even using the novel SSTR-ligand [^18^F]SiTATE. Some inter-reader variability was observed regarding the evaluation of uptake intensity prior to PRRT as well as compartment scoring of lymph nodes, indicating that those categories require special attention during further clinical validation and might be refined in a future SSTR-RADS version 1.1.

**Clinical relevance statement:**

SSTR-RADS 1.0 is a consistent framework for categorizing somatostatin receptor-targeted PET/CT scans when using [^18^F]SiTATE. The framework serves as a valuable tool for facilitating and improving the management of patients with NET.

**Key Points:**

- *SSTR-RADS 1.0 is a valuable tool for managing patients with NET*.
- *SSTR-RADS 1.0 categorizes patients with showing strong agreement across diverse reader expertise*.
- *As an alternative to [*^*68*^*Ga]-labeled PET/CT in neuroendocrine tumor imaging, SSTR-RADS 1.0 reliably classifies [*^*18*^*F]SiTATE-PET/CT*.

## Introduction

[^68^Ga]Ga-DOTATATE and [^68^Ga]Ga-DOTATOC are the commonly used ligands for the diagnostic work-up of neuroendocrine tumors (NET) with somatostatin receptor positron emission tomography/computed tomography (SSTR-PET/CT), and they serve as a highly sensitive, non-invasive imaging modality [1, 2]. These ligands, labeled with [^68^Ga], have shown high efficacy in detecting and visualizing neuroendocrine tumors (NET) and their metastases [3]. However, recent advances in clinical translation of the novel SSTR-ligand [^18^F]-Silicon-Fluoride-Acceptor (SiFA)-TATE ([^18^F]SiTATE) have brought significant advantages in the acquisition of SSTR-PET/CT scans for patients with NET: [^18^F] is a cyclotron-produced radionuclide with a half-life of 110 min, which provides a convenient time frame for synthesis, transportation, and in vivo distribution but is also short enough to avoid unnecessary radiation exposure for the patient [4, 5]. Moreover, challenges encompassing economic and logistic considerations of standard ^69^Ge/^68^Ga generators, along with small-scale production and comparatively short half-life of 68 min of [^68^Ga], can be successfully navigated. Compared to [^68^Ga]-radiolabeled tracers, [^18^F] radiotracers offer a lower positron energy with a higher image resolution, which is crucial for the detection of small tumor lesions, resulting in more sensitive and precise imaging [6, 7].

SSTR overexpression in NET forms the basis for the affinity of radiolabeled SSTR-analogs and makes NET lesions accessible not only for functional imaging but also for targeted therapy (peptide receptor radionuclide therapy, PRRT), a systemic treatment option in inoperable, metastatic NET patients [8, 9]. The NETTER-1 study, as reported by Jonathan Strosberg et al, provides the first long-term prospective results for the use of [^177^Lu]Lu-DOATATE (Luthera®) in treating locally advanced or progressive, well-differentiated, somatostatin receptor-positive midgut NET. A clinically relevant difference in median overall survival was observed with [^177^Lu]Lu-DOATATE, accompanied by a favorable long-term safety profile. These findings are significant, providing evidence of the efficacy of PRRT in a controlled trial for patients with progressive midgut NET. Future prospective studies utilizing combination therapy strategies are needed, emphasizing the importance of precisely selecting patients for PRRT to enhance overall survival outcomes [10, 11].

In recent years, the importance of standardized reporting has increased. A novel framework titled SSTR-RADS (Reporting and Data System) version 1.0 was proposed in 2018 for the standardized assessment of SSTR-PET/CT in patients with well-differentiated NET [12]. This five-point scale was introduced to serve as a tool for reliable interpretation and treatment planning of NET patients using SSTR-PET/CT. Previous studies showed promising results for the application of SSTR-RADS 1.0 in NET with high intra- and inter-reader agreement using standard ligands ([^68^Ga]Ga-DOTATATE and [^68^Ga]Ga-DOTATOC) [13, 14]. To evaluate the feasibility and robustness of this standardized framework in view of the recent introduction of [^18^F]-labeled SSTR-radioligands into clinical use [15], we aimed to determine the agreement between four readers with different levels of expertise in reading SSTR-PET/CT scans with the novel tracer [^18^F]SiTATE.

## Methods

### Study patients

Ninety-five consecutive patients with histologically confirmed NET who underwent [^18^F]SiTATE-PET/CT between 03/2020 and 04/2023 in a single tertiary cancer center were retrospectively identified. SSTR-PET/CT scans were conducted as part of the follow-up. Only patients with complete imaging data, a previous SSTR-PET/CT scan, and information about previous treatment available were included in the study. Most included patients (*n* = 92/95, 97%) had undergone treatment before PET/CT scans. The treatment depended on the individual patient and disease characteristics and involved different therapeutic approaches, such as surgery, somatostatin analogs, chemotherapy, locoregional procedures, tyrosine kinase inhibitors (TKI), everolimus, or radiotherapy. These therapies were administered either as an individual treatment or as a combination of different procedures. Patients who had received PRRT before undergoing PET/CT imaging were excluded from the analysis to ensure homogeneity of the study population. Table 1 provides an overview of patient characteristics.

**Table 1:** Patient characteristics

Characteristics		*n* = 95
Age (mean ± SD)	64 ± 13 y	
Sex	Female/male	43/52
Grading	G1	58
	G2	35
	G3	2
Primary tumor	GEP-NET	88
	Ileum/jejunum/mesenterial	63
	Pancreas	24
	Colon	1
	Non-GEP-NET	7
	Lung	3
	CUP (no primary tumor was detectable)	2
	Ovary	2
Metastases	Metastatic patients	89/95
	Liver	62
	Lymph node	49
	Soft tissue	58
	Skeleton	15
	Lung	6
Prior therapies	Patients pretreated	92/95
	Surgery	70
	Liver resection	16
	Somatostatin analog	68
	Chemotherapy	12
	Locoregional procedure	8
	Tyrosine kinase inhibitor (TKI)	1
	everolimus	4
	Radiation	5

*SD* standard deviation, *SSTR* somatostatin receptor, *G* grade, *GE*P gastroenteropancreatic, *NET* neuroendocrine tumor, *CUP* cancer of unknown primary

All patients gave written consent to undergo [^18^F]SiTATE PET/CT according to the regulations of the German Pharmaceuticals Act §13(2b). This study was performed in compliance with the principles of the Declaration of Helsinki and its subsequent amendments. The analysis of the data was approved by the institutional ethics board of LMU Munich (IRB 20-1077).

### [^18^F]SiTATE PET/CT imaging

PET/CT scans were acquired on Biograph 64 TruePoint w or TrueV and Biograph mCT Flow 20-4 R PET/CT scanners (Siemens, Healthcare GmbH) and were acquired 87  ± 14 min after intravenous administration of 225  ±  44 MBq [^18^F]SiTATE. Following the injection of intravenous contrast agent 1.5 times the body weight (Ultravist 300, Bayer Vital GmbH or Imeron 350 mg/mL, 2.5 mL/s, Bracco Imaging), diagnostic CT scans of the neck, thorax, abdomen, and pelvis (108–316 mAs; 100–160 kV) were acquired. PET was acquired with a 2.5 min per bed position. With CT scans serving for attenuation correction, PET images were reconstructed iteratively, with a transaxial 200 × 200 matrix using TrueX (including TOF, 2 iterations, and 21 subsets) with Gaussian post-reconstruction smoothing (2 mm full-width at half-maximum). SUVs were calculated using the patient’s body weight. Image analysis was performed using a dedicated software package (Hermes Hybrid Viewer, Hermes Medical Solutions). All acquired PET/CT scans were analyzed using dedicated software packages (syngo.via, Siemens Healthcare or Hermes Hybrid Viewer, Hermes Medical Solutions). Imaging reconstruction was automatically performed using built-in software. Three-millimeter slice reconstructions were used for reading. The synthesis of [^18^F]SiTATE represents a one-step strategy based on isotopic exchange using the SiFA-building block attached to the peptide, as described previously [16]. In the production of [^18^F]SiTATE, good manufacturing practice standards were applied. The production yield for [^18^F]SiTATE, began with an initial activity averaging 63 ± 9 GBq. Following synthesis, the average radiochemical yield was 53 ± 10%, subsequently corrected to 62 ± 11% after decay. The molar activities were determined to be 664 ± 145 GBq/μmol. Moreover, the reported radiochemical purity was 97 ± 0.9% [17].

### SSTR-RADS 1.0

An extensive definition of SSTR-RADS 1.0 is described in the original publication [3]. Lesions categorized as SSTR-RADS 1 are identified as definitively benign. SSTR-RADS 2 characterizes lesions displaying a minor level of SSTR-expression or non-specific uptake of radiotracer at an atypical site for NET, suggesting almost certainly benign lesions. SSTR-RADS 3 lesions necessitate further investigation (subsequent biopsy or follow-up imaging). These imaging findings are suggestive but not definitely NET. SSTR-RADS 4 comprises findings with enhanced SSTR expression at sites typical for NET lesions but lacking definitive findings in conventional imaging. SSTR-RADS 5 covers intense SSTR expression in locations characteristic of NET, supported by corresponding findings on conventional imaging.

### Readers

All scans were independently evaluated by two board-certified radiologists with over four years of experience in reading SSTR-PET/CT scans (experienced readers, ER1 and ER2) as well as one radiology resident and one nuclear medicine resident each with about two years of experience in reading SSTR-PET/CT scans (inexperienced readers, IR1 and IR2). All readers were masked to clinical data of the patients except for their age. All readers were familiar with the used workstations and software from clinical routine and were introduced to the SSTR-RADS 1.0 before the first read.

### Image interpretation

To assess inter-reader agreement, all four readers were advised to select a maximum of five target lesions (TLs) per scan, with no more than three TLs allocated to the same anatomical compartment. These lesions are identified based on two primary criteria to capture the most clinically significant findings within each scan. Firstly, TLs include findings that are either the most conspicuous—meaning they are clearly visible and distinguishable from surrounding tissues—or the largest in size on CT imaging. Secondly, TLs should also comprise lesions that demonstrate the highest tracer uptake on PET images. If more than five TLs can be detected, a dominant, representative lesion per “compartment” should be chosen [12]. This approach was adopted to ensure a consistent evaluation of the most clinically relevant lesions, facilitating an accurate comparison of reader interpretations. Predetermined organ compartments included the liver, lymph nodes (LNs), soft tissue (excluding LNs), skeleton, and lungs. An overall scan score was determined, which corresponds to the highest assigned score among all individual TLs.

Following the assignment of each TL to a specific SSTR-RADS score, readers evaluated the reasonability of PRRT for the patient based on the assigned scores and general image impression. Significant tracer uptake in the known tumor manifestations is a critical requirement as tracer enhancement greater than that in the liver suggests appropriate receptor expression [18]. To assess intra-reader agreement, all scans were reexamined by the four readers under identical conditions six weeks after the initial assessment.

### Statistical analysis

Continuous variables were represented as mean ± SD, and categorical variables were expressed as *N* (%). To assess the agreement of SSTR-RADS 1.0, the intraclass correlation coefficient (ICC) and their corresponding 95% confidence intervals (CIs) were applied. For evaluating both intra- and inter-reader agreement, we used Shrout & Fleiss, two-way random, consistency, multiple raters model in assessing rater reliability. Following Cicchetti’s criteria, ICC values below 0.40 were indicative of poor agreement, between 0.40 and 0.59 were considered fair, values ranging from 0.60 to 0.74 were regarded as good, and values equal to or greater than 0.75 were considered excellent [19]. Statistical significance was set at a *p* value less than 0.05. All statistical analyses were carried out using SPSS computer software (SPSS Statistics 29, IBM). Additionally, we compared the ICC agreements between two groups using “cocron” [20]. Comparison of proportions was performed using MedCalc Software Ltd. Comparison of proportions calculator (https://www.medcalc.org/calc/comparison_of_proportions.php; version 22.009; accessed September 12, 2023) [21].

## Results

### Inter-reader agreement

In two consecutive reads, the four readers selected 2609 lesions in 95 patients. A total of 153 lesions were selected by all four readers in the first read and 151 lesions in the second read. Subsequently, a comparison was made among these lesions. The distribution of the TLs among different compartments is shown in the supplementary Table S1. The inter-reader agreement was excellent, with an ICC of 91% and 85% in the initial and second reads, consecutively. When the assessment was performed individually for readers based on their experience, the inter-reader agreement was excellent: for the first reading, ERs achieved an ICC of 86%, and 79% in the second reading. IRs showed an excellent ICC of 94% for both reads (Table 2).

**Table 2:** Inter-reader agreement for the overall scan score for four identical TL among ER and IR

Inter-reader agreement ICC [95% CI]	Reader type		Organ system					All readers all organs
	ER	IR	Liver	LN	Soft tissue	Skeleton	Lung	
First read	0.864 [0.818; 0.898]	0.938 [0.919; 0.953]	0.831 [0.741; 0.895]	0.724 [0.559; 0.838]	0.932 [0.891; 0.960]	0.917 [0.812; 0.970]	NA	0.908 [0.882; 0.930]
Second read	0.789 [0.715; 0.844]	0.937 [0.917; 0.952]	0.850 [0.775; 0.904]	0.530 [0.241; 0.727]	0.740 [0.566; 0.855]	0.905 [0.785; 0.966]	NA	0.853 [0.810; 0.888]

*ICC* intraclass correlation coefficient, *CI* confidence interval, *ER* experienced reader, *IR* inexperienced reader, *LN* lymph nodes

#### Compartments

Among compartments, results were good to excellent with ICCs of ≥ 74% for liver, soft tissue, and skeleton in both reads as shown in Table 2. However, when it came to LN scoring, the results were less consistent, with an ICC of 72% for the first read and 53% for the second read. Analysis of the TL lung was not applicable due to a very low selection rate by all readers (*n* = 2 in both reads, respectively).

#### Overall scan score

**Fig. 1:**
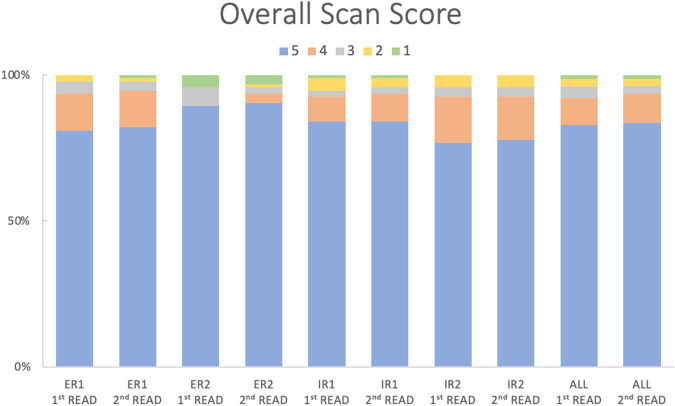
Distribution of SSTR-RADS for the overall scan score of ER and IR

For the overall scan score, ICCs among ERs were good to excellent (76% and 70%, respectively). ICCs of IRs showed excellent agreement, reaching 85% in the first read and 93% in the second read as depicted in Table 3. Out of the 95 SSTR-PET/CT scans, a significant majority was rated as SSTR-RADS 4 or 5 for the overall scan score by all readers as illustrated in Fig. 1.

**Table 3:** Inter-reader agreement for the overall scan score among experienced ER and IR

Inter-reader agreement ICC [95% CI]	Overall scan score		
	ER	IR	All readers
First read	0.760 [0.6439; 0.840]	0.852 [0.777; 0.901]	0.901 [0.865; 0.930]
Second read	0.698 [0.546; 0.799]	0.930 [0.895; 0.953]	0.918 [0.888; 0.942]

*ICC* intraclass correlation coefficient, *CI* confidence interval

#### Treatment decision for PRRT

**Fig. 2:**
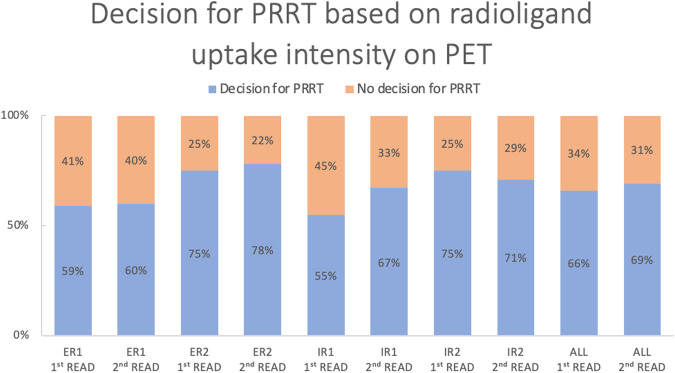
Treatment decision “functional imaging fulfills requirements for PRRT and qualifies patient as a potential candidate for PRRT” among ER and IR

All four readers were asked whether they would consider PRRT for each patient based on the assigned SSTR-RADS scores and the general image impression. Within the ER group, good agreement was attained for both reads, providing ICC values of 71% and 66%, respectively. Among IRs, the consensus was comparably good for the initial reading (ICC 66%) and the subsequent reading (ICC 70%). The agreement was excellent for both reads when comparing all four readers with an ICC of 86% and 85%, respectively (see Table 4). Overall, uptake intensity was rated sufficient for PRRT comparably often by ERs (*n* = 258) and IRs (*n* = 262; *p* = 0.850). A significant inconsistency was noted between the recommendation of ER1 who judged uptake to be sufficient for PRRT in 59% (*n* = 56) and 60% (*n* = 57; *p* = 0.020) of cases in both reads and ER2 in 75% (*n* = 71) and 78% (*n* = 74; *p* = 0.008) for the first and second read, respectively (see Fig. 2). Similar findings were observed when IR1 rated uptake intensity sufficient for PRRT in 55% (*n* = 52) and IR2 in 75% (*n* = 71; *p* = 0.004) of all cases in the first read, whereas IR1 (*n* = 64) and IR2 (*n* = 67; *p* = 0.670) suggested PRRT equally often in the second read.

**Table 4:** Inter-reader agreement on the decision for PRRT among ER and IR

Inter-reader agreement ICC [95% CI]	Decision for PRRT		
	ER	IR	All readers
First read	0.705 [0.5657; 0.804]	0.657 [0.484; 0.771]	0.856 [0.802; 0.898]
Second read	0.658 [0.486; 0.772]	0.697 [0.548; 0.798]	0.849 [0.792; 0.893]

*ICC* intraclass correlation coefficient, *CI* confidence interval

### Intra-reader agreement for compartments, overall scan score, and decision for PRRT

**Fig. 3:**
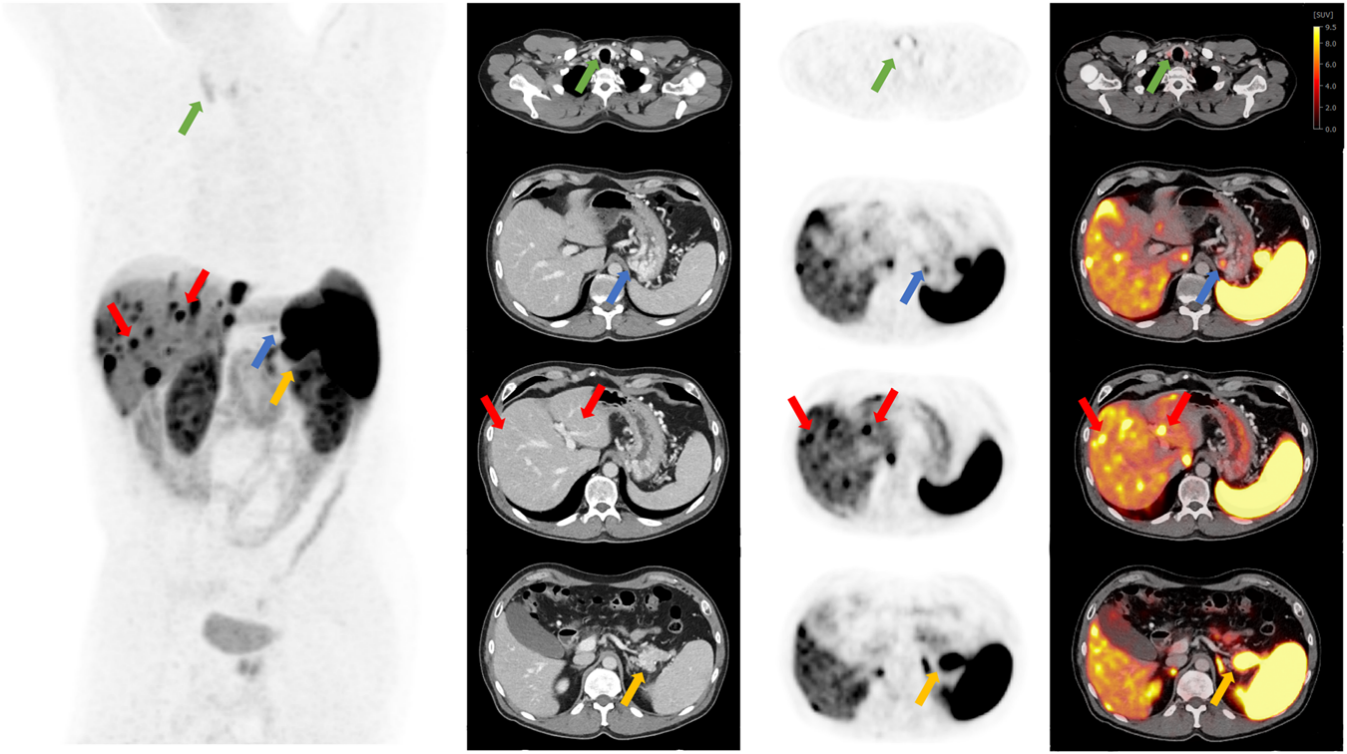
Detailed PET/CT images from a 55-year-old man with a known neuroendocrine tumor of the pancreas (G1, Ki-67 1–2%), diagnosed two years ago. He received systemic chemotherapy (Capecitabin/ Temozolomid and FOLFOX) and now presented with stable disease under therapy with Somatuline. The patient received contrast-enhanced diagnostic CT. In the first row, the thyroid gland and in the second to fourth image series, the upper abdomen is depicted. From left to right: MIP (Maximum Intensity Projection), CT component, PET component, fused CT and PET images of [^18^F]SiTATE PET/CT-scan. In the first row, [^18^F]SiTATE PET shows low uptake in the thyroid gland with no suspect finding on the axial CT, classified as SSTR-RADS 1 (green arrow). In the subsequent row, a lesion in the cardia of the stomach with no suspect finding on the axial CT was classified by one experienced reader and one inexperienced reader as SSTR-RADS 3 A (blue arrow) “Suggestive of, but not definitive for, NET—further workup might be required”. The patient received a gastroscopy, which attested to a non-erosive gastroesophageal reflux disease. [^18^F]SiTATE PET showed intensive uptake in the liver, but three readers could not identify any corresponding finding on CT, so this lesion was classified SSTR-RADS 4, except for one reader, who categorized this lesion as SSTR-RADS 5 (red arrow). Intense uptake in the pancreas with corresponding findings on CT can be found in the last row on axial CT, [^18^F]SiTATE PET, and fused PET/CT, so this lesion was classified SSTR-RADS 5 by all four readers (yellow arrow). All readers rated the uptake intensity as sufficient for recommending PRRT according to the SSTR-RADS 1.0 criteria

The agreement within an individual reader was excellent for scoring compartments, overall scan score, and the decision for PRRT with ICCs exceeding 90% in both reads, as presented in Table 5. A patient example with assigned SSTR-RADS scores is presented in Fig. 3.

**Table 5:** Intra-reader agreement on organ system-/target lesion-based, overall scan score and decision for PRRT scoring among ER and IR

	Reader type			Organ system							All readers all organs
	ER	IR		Liver	LN		Soft tissue	Skeleton		Lung	
Intra-reader agreement ICC [95% CI]	0.965 [0.957; 0.971]	0.993 [0.992; 0.994]		0.980 [0.974; 0.984]	0.948 [0.932; 0.960]		0.992 [0.990; 0.993]	0.974 [0.962; 0.982]		0.977 [0.944; 0.991]	0.988 [0.986; 0.989]

Intra-reader agreement ICC [95% CI]			Overall scan score								
			ER			IR			All readers		
			0.921 [0.895; 0.941]			0.949 [0.932; 0.962]			0.935 [0.920; 0.947]		

Intra-reader agreement ICC [95% CI]			Decision for PRRT								
			ER			IR			All readers		
			0.923 [0.897; 0.942]			0.897 [0.863; 0.923]			0.910 [0.890; 0.92]		

*ICC* intraclass correlation coefficient, *CI* confidence interval, *LN* lymph nodes

## Discussion

These results indicate that the novel [^18^F]-labeled SSTR-radioligand [^18^F]SiTATE has high clinical potential to replace the commonly used ligands [^68^Ga]Ga-DOTATATE and [^68^Ga]Ga-DOTATOC for PET/CT imaging for NET due to its superior clinical applicability, including the potentially superior availability, cost efficiency, and physical properties [4, 22]. The novel framework for the standardized interpretation of SSTR-PET/CT and treatment planning of NET patients titled SSTR-RADS 1.0 has already been assessed with [^68^Ga]Ga-DOTATOC and achieved excellent inter- and intra-reader agreements [13, 14]. This study aimed to assess the reliability of SSTR-RADS 1.0 and validate the reproducibility and robustness of the standardized reporting system using the novel tracer [^18^F]SiTATE in an extensive multi-reader study.

The application of SSTR-RADS 1.0 on [^18^F]SiTATE PET/CT scans obtained consistently good to excellent inter-and intra-reader agreement for the overall scan score in the first and second read (ICC ≥ 70%), although the ICC for the overall scan from previously mentioned studies was superior (ICC ≥ 85% and ICC 88%). This agreement was supported by an excellent inter- and intra-reader agreement even among less ER (ICC ≥ 85%) matching previous results (ICC ≥ 85%), confirming the high reproducibility of SSTR-RADS for all readers, which is essential to provide the clinician with reliable information. In some cases, IR demonstrates better agreement than ER. One possible explanation is that IR may have been exposed to more recent or uniform training protocols. This consistency in their training could result in them adhering more closely to the guidelines, leading to a higher level of agreement among them. Another factor to consider is the susceptibility of ER to cognitive biases. Their prior experiences might influence them to make assumptions or interpretations that do not align with the guidelines (SSTR-RADS 1.0). In contrast, IR, perhaps adhering more strictly to the given guidelines, tend to show higher agreement rates among themselves. Furthermore, the cautious approach taken by IR, who are conscious of their limited experience, may also play a role. Their strict adherence to the guidelines, motivated to minimize errors, likely results in greater consistency in their evaluations. ER, feeling more confident in their judgments, might be more inclined to rely on personal assessments and deviate from the guidelines, which could lead to discrepancies in their evaluations. These factors highlight the complex interplay between experience, training, and cognitive biases in influencing reader agreement. It suggests that while experience brings valuable insights, it also introduces variables that can affect the consistency of evaluations, particularly in contexts requiring adherence to specific guidelines such as SSTR-RADS 1.0. Given the theranostic approach for NET patients, an accurate overall scan score assessment is crucial for selecting eligible patients for PRRT [23]. This study demonstrates excellent inter-reader agreement among all four readers in both reads (ICC ≥ 66%) and excellent intra-reader agreement (ICC ≥ 90%), in accordance with the previous study by Grawe et al with an inter-reader agreement of ICC ≥ 66% und intra-reader agreement of 87%. However, we observed that ER2 considered PRRT more often (1st read: *n* = 71, 2nd read: *n* = 74) than ER1 (1st read: *n* = 56, 2nd read: *n* = 57), which is in line with previously published data on significantly varying results considering PRRT, stating decision-making for PRRT seems to require experience and training [13, 14]. In contrast to the above-mentioned previous studies, our analysis showed that ER (*n* = 258) and IR (*n* = 262) recommended PRRT equally often. Both SSTR-RADS 4 and 5 imply a high SSTR-expressing tumor burden, which is needed for an informed decision on PRRT [24]. In our study, the majority of patients (83%) were rated with an overall scan score of 4 or 5; therefore, an excellent inter-reader agreement could be identified with an ICC of 86% in the first and 85% in the second read. However, even when patients have the same overall scan score assigned by all four readers, a consistent consideration of PRRT based on imaging may not always be offered. The variation in how tumor burden was assessed both by ERs and IRs results in a significant difference in the frequency of deciding on PRRT. While assessing the use of PRRT can be complex, a good inter-reader agreement is still demonstrated. (ICC for the 1st read: 71%, ICC for the 2nd read 66%). Our study showed excellent agreement among readers, both between different readers and the same reader over time, for patients with high overall scan scores (4 or 5), which suggests a significant overexpression of somatostatin receptors. However, deciding to proceed with PRRT is not based solely on the overall scan score. It’s important to note that a high scan score while indicating overexpression, does not automatically qualify a patient for PRRT. Moreover, the choice between scores 4 and 5 does not significantly impact the decision on PRRT, as the lesions exhibit almost equivalent SSTR uptake. For example, a patient might have an overall scan score of 4 or 5 according to SSTR-RADS 1.0 but only one small metastasis, indicating that PRRT might not be the best treatment option due to the low disease burden. No reader recommended PRRT for patients with scores from 1 to 3, highlighting that a score of 4 or 5, signaling significant receptor overexpression, is crucial in considering this treatment. This score is also vital in effectively identifying patients for whom PRRT might not be suitable, due to a lower likelihood of significant disease. In conclusion, the decision of whether PRRT is suitable for a patient or not is complex and versatile. It is recommended to involve a multidisciplinary team (MDT) in this process, as the decision for PRRT never solely relies on imaging characteristics as assessed by reporting framework systems [8]. The MDT meeting should consider various factors during their discussion, including detailed patient history, tumor characteristics (such as size and growth patterns), the tumor’s primary location, and its grade [25]. Thus, we propose that the standardized system SSTR-RADS 1.0 can play a supportive role in assessing NET as potential candidates for PRRT, even among fewer ERs. The development and improvement of standardized reporting frameworks for various entities, including prostate cancer, such as PSMA-RADS version 2.0 and PROMISE V2, represents a substantial advancement in the field of medical imaging and clinical decision-making [26]. These frameworks are designed to enhance the accurate characterization of lesions and disease extent. Furthermore, PROMISE V2 suggests a response monitoring framework, specifying both qualitative and quantitative parameters to determine response according to PSMA-PET progression and Response Evaluation Criteria in PSMA-PET/Computed Tomography [27]. The absence of a dedicated response monitoring framework for SSTR-PET/CT imaging represents a notable gap in current clinical management and follow-up of patients with NET and offers an opportunity for future advances in this area.

The compartment-based assessment of the SSTR-RADS scans showed excellent inter-reader agreements among all readers for liver, soft tissue, and skeleton (ICC ≥ 74%), although there are some differences in biodistribution between [^18^F]- and [^68^Ga]-labeled SSTR-PET/CT, such as higher physiological uptake of [^18^F]SiTATE in adrenal glands, spleen, and liver, but lower physiological uptake in the thyroid gland, lungs, and bones [28]. In the assessment of LN scoring, the inter-reader agreement varied between good (ICC 72%) in the first read and fair (ICC 52%) in the second read, even though the intra-reader agreement of LN was 95%, which is comparable with an ICC of 76%, 50%, and 95% in the previous study. Most of the LNs were scored with SSTR-RADS 4 or 5, indicating intense uptake at sites typical for NET, but facing difficulties in evaluating small TLs such as LNs in anatomical imaging (CT) since conventional anatomic imaging often fails to differentiate benign from metastatic LNs [29]. Small LNs could potentially be overlooked or be mistaken for other structures due to their small size. Furthermore, image or motion artifacts can affect the accuracy of lymph node assessment. Therefore, it is essential to consider these factors and rely on a combination of clinical context, imaging characteristics, and additional imaging modalities such as PET to accurately assess small LNs in NET patients and underline the importance of functional imaging.

In our study, the ICC for inter-reader agreement in lung lesions could not be conducted due to the limited number of lung lesions being selected by the readers (*n* = 2 in both reads, respectively). Suspect lung lesions were identified in only two patients in both reads by all four readers. This finding is consistent with the relatively rare occurrence of lung carcinoids [30]. A previous study showed that a significantly higher tumor uptake was described in almost all tumor lesions in common metastatic sites of NET, including the liver, LNs, and bone, except for lung lesions [28]. This could result in lung lesions being chosen less frequently. In our study, the selection of TLs was guided by two primary criteria designed to capture the most clinically significant findings within each scan. The first criterion was the conspicuity and size of lesions on CT imaging and the second criterion focused on lesions demonstrating the highest tracer uptake on PET images. Regarding lung metastases, these lesions are known to be relatively challenging to recognize due to factors such as their size, location, and the potential for confounding findings in the lung, like benign nodules or inflammatory changes. In our study, despite the guidelines for identifying TLs, it was observed that in only two of the six cases with suspected lung nodules, the readers described these nodules as suspect in their evaluation. This highlights the inherent challenges in detecting and interpreting lung metastases or lung carcinoids, even with advanced imaging techniques and standardized evaluation criteria. It is crucial for continued research on NET patients to prioritize selecting lung lesions in NET patients to better understand their behavior and response to treatment approaches. Research should focus on unraveling the underlying reasons behind the varying tumor uptake of [^18^F]SiTATE in lung lesions compared to other metastatic sites in tumors.

Some liver metastases, as can also be seen in Fig. 3, are assigned a score of only four due to a lack of CT correlation, despite being definitively NET metastases. This emphasizes the significant role of molecular (PET) imaging in assessing patients with NET. The spatial resolution of PET/CT in the subcentimeter range is a known limitation of this imaging method. Especially for small liver lesions or metastases, a dedicated MRI of the liver plays an important role and should be performed in all patients with suspected liver metastases. In these cases, access to PET/MRI would be of great additional benefit [31].

In reviewing the imaging findings in accordance with the SSTR RADS 1.0, it is important to highlight that a classification of SSTR RADS 5 is typically reserved for lesions with a high suspicion of metastasis, strengthened by CT correlation.

Several limitations exist in this study including its retrospective design. There is no histopathological comparison available for each target lesion, which can restrict a more comprehensive assessment of the given findings. For lesions classified as SSTR-RADS 4 or 5, the specific histopathologic finding of these lesions is less pivotal, as they are almost certainly indicative of NET or metastases. It can be assumed that the vast majority of lesions were true positives, so histologic validation would have limited impact and would carry the risk of sampling error. Additionally, the blinding of readers to patients’ clinical status, while intended to maintain objectivity, may have unintentionally influenced inter- and intra-reader agreement. With an improved understanding of the clinical context, agreement among readers may potentially increase even further, emphasizing the importance of integrating clinical information into the interpretation process of [^18^F]SiTATE-PET/CT scans.

Given these findings, it appears reasonable to apply the SSTR-RADS for characterizing individual lesions identified in SSTR-PET/CT, considering the substantial consensus not only in the overall scan score but also in the case of individual lesions. Several standardized reporting systems have been proposed for a variety of imaging modalities and tumor entities. These include RADS for imaging of the breast (BI-RADS), prostate (PI-RADS), lung (LUNG-RADS), liver (LI-RADS), and thyroid (TI-RADS) [32]. SSTR-RADS was introduced in adaption to establish standardized systems using a five-point scale. Application of the SSTR-RADS is recognized as a simple and easily understandable tool, as all readers were able to familiarize with the SSTR-RADS in a very short time. Implementation into the clinical routine can be achieved without significant additional effort [33]. [^18^F]SiTATE serves as a promising new radiopharmaceutical for PET imaging of NET [28], demonstrating its potential in the field of NET. However, it has also proven to be a valuable tool for identifying meningiomas. A recently published study has indicated that the novel PET ligand [^18^F]SiTATE, which targets SSTR in meningioma patients, demonstrated excellent contrast against healthy structures and non-meningioma lesions, while also effectively detecting osseous extensions and previously unidentified meningioma lesions [34]. Consequently, [^18^F]SiTATE-PET/CT gains importance in accurate NET diagnosis and treatment decisions, as a comparable tumor-to-background ratio and even higher tumor-to-liver ratio than that of the gold standard [^68^Ga]Ga-DOTATOC were detected [35]. The favorable characteristics of [^18^F] and the kit-like labeling of [^18^F]SiTATE enable improved logistics and diagnostic possibilities [15].

## Conclusion

Our study demonstrated the robustness and reproducibility of SSTR-RADS 1.0 for categorizing SSTR-PET/CT of NET patients using the novel ligand [^18^F]SiTATE, demonstrating high agreement among readers with varying levels of experience and within the same reader. The presented framework serves as a valuable tool for facilitating and improving the management of NET patients within the clinical setting by establishing diagnostic and treatment planning standards. However, in line with previously published data, accurate assessment of LNs remains challenging, and treatment decisions should take clinical and pathological findings into account.

## Supplementary information

ELECTRONIC SUPPLEMENTARY MATERIAL

## Abbreviations

CI: Confidence Intervals
CT: Computed tomography
DOTA-TATE: DOTA(0)-Phe(1)-Tyr(3))octreotate
DOTA-TOC: DOTA(0)-Phe(1)-Tyr(3))octreotide
ER: Experienced reader
GMP: Good manufacturing practice
ICC: Intraclass correlation coefficient
IR: Inexperienced reader
LN: Lymph node
MDT: Multidisciplinary team
MRT: Magnetic resonance tomography
NET: Neuroendocrine tumor
PET: Positron emission tomography
PRRT: Peptide receptor radionuclide therapy
PSMA: Prostate-specific membrane antigen
RADS: Reporting and Data System
SiTATE: Silicon fluoride acceptor tagged Tyr(3)octreotate
SSTR: Somatostatin receptor
SUV: Standard Uptake Value
TL: Target lesion
