# Supplementary material for: Validation of the standardization framework SSTR-RADS 1.0 for neuroendocrine tumors using the novel SSTR‑targeting peptide [18F]SiTATE
Source: Eur Radiol. 2024 May 20;34(11):7222–32. doi: 10.1007/s00330-024-10788-3 (PMC11519286; doi:10.1007/s00330-024-10788-3)
Supplement: Supplementary file 1 — ELECTRONIC SUPPLEMENTARY MATERIAL [file 330_2024_10788_MOESM1_ESM.pdf]

Validation of the standardization framework SSTR-RADS 1.0 for neuroendocrine tumors using the novel SSTR-targeting peptide [18F]SiTATE
ELECTRONIC SUPPLEMENTARY MATERIAL

Table S1 Distribution of the target lesions (TL) among the five different compartments in the first and second read

	**Liver**	**Lymph node**	**Soft tissue**	**Skeleton**	**Lung**
**1st read**	52/153 (34%)	43/153 (28.1%)	42/153 (27.4%)	14/153 (9.2%)	2/153 (1.3%)
**2nd read**	58/151 (38.4%)	43/151 (27.2%)	36/151 (23.8%)	14/151 (9.3%)	2/151 (1.2%)
